# Firework-related ocular trauma: a comment

**DOI:** 10.5935/0004-2749.2023-0322

**Published:** 2024-04-03

**Authors:** Hinpetch Daungsupawong, Viroj Wiwanitkit

**Affiliations:** 1 Private Academic Consultant, Phonhong, Lao People’s Democratic Republic; 2 Department of Pharmaceutical Sciences, University Centre for Research and Development, Chandigarh University, Mohali, India

Dear Editor,

We would like to discuss the publication “Firework-related ocular trauma in Pernambuco, Brazil”^(1)^. The medical records of patients with firework-related trauma who were admitted to emergency rooms between January 2012 and December 2018 were reviewed in this retrospective study. Patient demographics, nature of the injuries, and type of treatment received were among the information gathered. However, it was necessary to analyze the final visual acuity and origin of patients who were followed for >30 days. The study included 314 patients, and 370 eyes were evaluated. The majority of patients were male (79.0%), and 51.0% of them were from the metropolitan region of Recife. The average age of the patients was 25.6 years, and 17.8% of them had bilateral involvement. The month with the highest number of cases was June (48.4%), and the most commonly affected sites were the eyelids (24.6%) and ocular surface (68.1%). Surgical treatment was required in 23.5% of the eyes. After treatment, 10.0% of the eyes exhibited a final visual acuity of 20/400, and 91.9% of the eyes were from patients from the countryside or another state. Patients from the countryside had a higher risk of developing firework-related blindness than those from the metropolitan area (odds ratio, 5.46). One of the study’s weaknesses is its retrospective design, which makes it more difficult to account for confounding factors and increases the risk of bias. Furthermore, the study only included patients who were admitted to emergency rooms, which might have excluded less serious cases that received care in other departments. In addition, the study did not evaluate outcomes for >30 days, which may have limited our understanding of the full impact of firework-related trauma. Future prospective studies that collect more reliable data and enable improved control over confounding variables are required. Furthermore, extending the duration of follow-up beyond 30 days may yield significant information about the consequences and complexities of fireworks-related injuries. Preventive measures and focused interventions may be benefited from more studies into variables influencing patients from rural areas, who are at a higher risk of developing blindness.
